# Peroxisome Proliferator-Activated Receptor (PPAR) γ and PPARα Agonists Modulate Mitochondrial Fusion-Fission Dynamics: Relevance to Reactive Oxygen Species (ROS)-Related Neurodegenerative Disorders?

**DOI:** 10.1371/journal.pone.0064019

**Published:** 2013-05-13

**Authors:** Juan M. Zolezzi, Carmen Silva-Alvarez, Daniela Ordenes, Juan A. Godoy, Francisco J. Carvajal, Manuel J. Santos, Nibaldo C. Inestrosa

**Affiliations:** 1 Departamento de Biología, Facultad de Ciencias, Universidad de Tarapacá, Arica, Chile; 2 Centro de Envejecimiento y Regeneración (CARE), Facultad de Ciencias Biológicas, Pontificia Universidad Católica de Chile, Santiago, Chile; 3 Departamento de Biología Celular y Molecular, Facultad de Ciencias Biológicas, Pontificia Universidad Católica de Chile, Santiago, Chile; Federal University of Rio de Janeiro, Brazil

## Abstract

Recent studies showed that the activation of the retinoid X receptor, which dimerizes with peroxisome proliferator-activated receptors (PPARs), leads to an enhanced clearance of Aβ from the brain of transgenic mice model of Alzheimer’s disease (AD), because an increased expression of apolipoprotein E and it main transporters. However, the effects observed must involve additional underlying mechanisms that have not been yet explored. Several studies conducted in our laboratory suggest that part of the effects observed for the PPARs agonist might involves mitochondrial function and, particularly, mitochondrial dynamics. In the present study we assessed the effects of oxidative stress challenge on mitochondrial morphology and mitochondrial dynamics-related proteins in hippocampal neurons. Using immunofluorescence, we evaluated the PPARγ co-activator 1α (PGC-1α), dynamin related protein 1 (DRP1), mitochondrial fission protein 1 (FIS1), and mitochondrial length, in order to determine if PPARs agonist pre-treatment is able to protect mitochondrial population from hippocampal neurons through modulation of the mitochondrial fusion-fission events. Our results suggest that both a PPARγ agonist (ciglitazone) and a PPARα agonist (WY 14.643) are able to protect neurons by modulating mitochondrial fusion and fission, leading to a better response of neurons to oxidative stress, suggesting that a PPAR based therapy could acts simultaneously in different cellular components. Additionally, our results suggest that PGC-1α and mitochondrial dynamics should be further studied in future therapy research oriented to ameliorate neurodegenerative disorders, such as AD.

## Introduction

Alzheimer’s disease (AD) is an age-associated neurodegenerative disorder characterized by progressive memory loss and cognitive impairment [1], [2]. Brain atrophy and the gradual loss of neurons occur mainly in the hippocampus, frontal cortex and limbic areas. Senile plaques formation, derived from amyloid-β-peptide (Aβ) accumulation in the brain, is a pathologic hallmark of AD [3]. Until now, no effective treatment is available to stop or reverses this devastating disease.

Recently, Cramer et al. [4] found that treatment with an old drug, bexarotene, reverses the cognitive deficit observed in a mice model of AD. Bexarotene, a retinoid related molecule, is an anti-neoplastic drug, approved by the FDA (*U.S. Food and Drug Administration*) and the EMA (*European Medicines Agency*), for the treatment of some types of skin cancer [5], [6]. Cramer et al. [4] exploited the retinoid-based properties of bexarotene to activate the retinoid receptor (RXR) [5], inducing apolipoprotein E (ApoE), ABCA1 and ABCG1 expression, leading to enhanced Aβ clearance from the brain. However, it is well recognized that ApoE4 is an increased-risk related ApoE isoform, and patients, who carries this variant, could actually not obtain any benefit, or worst, exhibits an even greater risk to develop AD or any other neuropathology [1], [2], [3].

On the other hand, the mechanism proposed by Cramer [4] and others [7], [8] fails to explain the wide range of effects observed after bexarotene treatment. Indeed, these studies offers a convincing explanation about Aβ clearance from the brain, but fails to explain the cognitive recovery observed, considering the neuronal loss and the general damage derived from Aβ accumulation. In fact, it has been established that Aβ alters synaptic function and neurotransmission by interacting with both, synaptic ion channels and mitochondria [9]–[13]. Moreover, several authors have demonstrated that Aβ also alters the neuronal metabolism due to induction of mitochondrial dysfunction which contributes to oxidative damage because an increased production of mitochondrial reactive oxygen species (ROS) [14]–[16]. In fact, *in vitro* experiments have confirmed that one of the main neurotoxic mechanisms of Aβ aggregates is through oxidative stress [3], [10], [17], [18]. Recently, mitochondrial dynamics, which considers fusion-fission events, has been described as a critical mechanism related with mitochondrial and cellular fate after critical insults, mainly because successive fusion-fission cycles allows to eliminate dysfunctional organelles and to repair the mitochondrial DNA that could ended damaged after a toxic challenge [19].

Since a decade ago, we have studied Nuclear Receptors (NR), particularly the peroxisome proliferator activated-receptors (PPARs) [20]–[24]. PPARs, a type II NR, are characterized by dimer formation with the RXR [25]. Activation of RXR:PPAR requires removal of transcriptional co-repressors and addition of co-activators, such as peroxisome proliferator-activated receptor γ co-activator 1 α (PGC-1α) [25], [26]. Moreover, PGC-1α has been described as highly expressed in the brain [26] and, recently, it has been demonstrated that AD brain exhibits decreased expression of PGC-1α [27]. Further investigations have leaded to considers PGC-1α as a critical transcriptional co-activator linked to cellular metabolism and mitochondrial biogenesis, and which abnormal expression could further account for the altered neuronal metabolism and diminished mitochondrial density often reported in several AD models [27], [28]. Recent research carried out in our laboratory has demonstrated that WY 14.643, a PPARα agonist, improves significantly the cognitive abilities and reduces neuronal damage in a mouse model of AD [24]. These findings have prompted us to suggest that part of the effects observed after WY treatment might be due to an underlying mechanism which involves a PGC-1α-related response.

In the present study we have evaluated the impact of oxidative stress challenge and PPAR-agonists treatment on mitochondrial morphology and on the PGC-1α-related mitochondrial fusion-fission events on hippocampal neurons. The scope of this research is not only to complement the actual knowledge regarding PPARs-mechanisms of action, but also to propose mitochondrial dynamics as an additional target for future oxidative stress-related neurodegenerative disorders therapies.

## Materials and Methods

### Ethics Statement

Sprague-Dawley rats used in these experiments were housed at the Faculty of Biological Sciences of the P. Universidad Catolica de Chile and handled according to guidelines outlined and approved by the Institutional Animal Care and Use Committee (IACUC) at the Faculty of Biological Sciences of the P. Catholic University of Chile; and in accordance with the main directions from the Science and Technology National Commission (CONICYT) about experimental animal research. Animals were euthanized by anesthesia overdose. In our case, the IACUC did not require approval of specific protocols, because housing and handling procedures, as well as euthanasia, are well standardized protocols conceived and constantly monitored by IACUC itself. No experimental procedures were performed on the rats prior to euthanasia.

### Hippocampal Neuronal Cultures

Mixed 18 days embryonic Sprague-Dawley rat hippocampi were dissected, and primary hippocampal cultures were prepared as described previously [29]. Hippocampal cells were seeded in polylysine-coated wells and cultured in Neurobasal medium (Invitrogen Corp., Carlsbad, CA), supplemented with B27 (Invitrogen). 11 DIV cultured hippocampal neurons were used for several experiments. 2 µM 1-β-D-arabinofuranosylcytosine (Ara-C, Cat. N°C-6645, Sigma-Aldrich, St. Louis, MO) was used to reduce the glial content in the culture.

### Ciglitazone (PPARγ agonist)-H_2_O_2_ Challenge Assay

Hippocampal neurons (11 DIV) were pre-treated for 2 h with 10 µM ciglitazone (CIG) (Sigma Aldrich) prior to 30 and 60 min of 25 µM H_2_O_2_ challenge. Additionally, 5 µM GW9662 (PPARγ inhibitor) (Sigma Aldrich) was used as control. At the end of experimental time, neurons were washed and labeled with 50 nM Mitotracker Red (Molecular Probes, Eugene, OR) for 30 min. at 37°C. Cells were then fixed by paraformaldehyde-sucrose 4% fixative and permeabilized with PBS-Triton X 0.01%. Immunofluorescence images were captured with a Zeiss LSM 5 Pascal confocal microscope. Mitochondrial length was assessed using Image J software by Feret’s diameter analysis. To allow comparison, mitochondrial length was classified into three different categories ranging from 0.21 to 0.54 µm (small), 0.55 to 1.59 µm (medium); and 1.60 to 10 µm (large). A minimum of 10 microphotographs were recorded for each treatment (per replicate) and scored.

### WY (PPARα agonist)-H_2_O_2_ Challenge Assay and PGC-1α Assessment

Hippocampal neurons (11 DIV) were pre-treated for 12 h with 50–100–150 µM WY 14.643 (WY) (Aptuit, Lenexa, KA) solution prior to 30–60–120 min of 25 µM H_2_O_2_ challenge. At the end of experimental time, neurons were washed and labeled with 50 nM Mitotracker Red (Molecular Probes) for 30 min at 37°C. Cells were then fixed by paraformaldehyde-sucrose 4% fixative and permeabilized with PBS-Triton X 0.01%. Hippocampal neurons were additionally stained for PGC-1α (anti-PGC1α polyclonal antibody ab54481, 1∶300, Abcam Inc., Cambridge, MA) and incubated over-night at 4°C. After primary antibody incubation cells were washed and incubated with anti-mouse IgG- Alexa Fluor-488 (1∶1000) (Invitrogen) as secondary antibody for 1 h at 37°C. Immunofluorescence images were captured with a Zeiss LSM 5 Pascal confocal microscope. Mitochondrial length and PGC-1α signal intensity were assessed using Image J software by Feret’s diameter analysis and Corrected Total Cell Fluorescence (CTCF), respectively. To allow comparison, mitochondrial length was classified as for CIG.

### Mitochondrial Dynamics Assay

Hippocampal neurons (11 DIV) were pre-treated for 12 h with 150 µM WY (Aptuit) solution prior to 120 min of 6.25–12.5–25–50–100 µM H_2_O_2_ challenge. At the end of culture time, neurons were washed and labeled with 50 nM Mitotracker Red (Molecular Probes) for 30 min at 37°C. Cells were then fixed by paraformaldehyde-sucrose 4% fixative and permeabilized with PBS-Triton X 0.01%. Hippocampal neurons were additionally stained for dynamin related protein 1 (DRP1) (mouse anti-DLP1, BD Bioscience, San José, CA) and mitochondrial fission protein 1 (FIS1) (rabbit anti-Fis1, Enzo Life Science, Flamingdale, NY), and incubated over-night at 4°C. After primary antibody incubation cells were washed and incubated with anti-mouse IgG-Alexa Fluor-633 (1∶500) (Invitrogen) and anti-rabbit IgG-Alexa Fluor-488 (1∶100) (Invitrogen) respectively, as secondary antibodies for 1 h at 37°C. Immunofluorescence images were captured with a Zeiss LSM 5 Pascal confocal microscope. Image analysis was performed using “Find Maxima” tool in Image J software, complemented with Menders co-localization analysis [30], which allows to finds maximal fluorescence points within each microphotograph. Each point will corresponds to the overlap of the three color channels considered in the study. Vasopressin was included as a positive control.

### Statistical Analysis

Results are presented as means ± standard error (mean ± s.e.m) and normalized against controls. One-way ANOVA followed by Bonferroni’s post hoc test, was used to establish statistically significant differences among treatments. Two-way ANOVA followed by Bonferroni’s post hoc test, was used to assess statistically significant differences among treatment types and duration. Analysis and plots were done using GraphPad Prism (GraphPad Software Inc., La Jolla, CA).

## Results

### CIG, a PPARγ agonist, Prevents Mitochondrial Size Reduction in Hippocampal Neurons Induced by H_2_O_2_ Challenge

**Figure 1:**
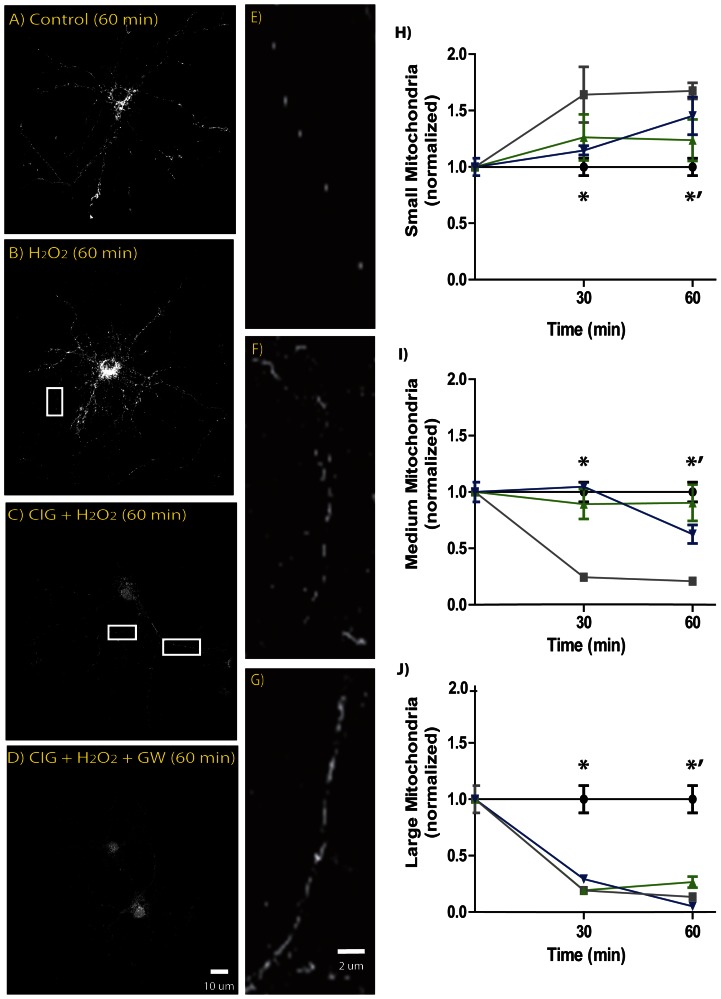
H_2_O_2_-induced mitochondrial size decrease is prevented by CIG pre-treatment in Hippocampal neurons. A, B, C and D, are representative microphotographs of mitochondrial population under different experimental conditions. E, F, G: image enlargement to show the variations in the mitochondrial morphology observed under different experimental conditions. H, I, J: Normalized mitochondrial size quantification (black: control; gray: H_2_O_2_; green: CIG+H_2_O_2_; blue: CIG+H_2_O_2_+ GW). H) * and *’ indicate statistically significant differences between H_2_O_2_ and Control (*: F 6.542, d.f. 3, C.I. 0.07389 to 0.6266, t 3.856, p<0.001; *’: C.I. 0.08504 to 0.6519, t 3.955, p<0.001); I) * and *’ indicate statistically significant differences between H_2_O_2_ and Control (*: F 17.8, d.f. 3, C.I. −0.3665 to −0.1151, t 5.829, p<0.001; *’: C.I. −0.3805 to −0.1227, t 5.937, p<0.001). J) * and *’ indicate statistically significant differences between Control and all other experimental conditions (*: F 18.99, d.f. 3. H_2_O_2_ C.I. −0.1696 to −0.04917, t 5.527, p<0.001; H_2_O_2_+ CIG C.I. −0.1708 to −0.04728, t 5.371, p<0.001; *’: H_2_O_2_ C.I. −0.1787 to −0.05512, t 5.757, p<0.001; H_2_O_2_+ CIG C.I. −0.1595 to −0.03905, t 5.015, p<0.001).

Considering oxidative stress as a critical pathway in the pathophysiology of several neurodegenerative disorders, such as the Aβ-mediated neurotoxicity in AD [15]–[17], we exposed hippocampal neurons to 25 µM H_2_O_2_ challenge for 30 and 60 min. As we have previously demonstrated [21], at this concentration H_2_O_2_ is able to induce neurotoxicity but without affecting severely the total neuronal number, allowing better comparison between treatments. As expected, H_2_O_2_ challenge induced a statistically significant alteration of the dynamic process of mitochondrial population, with an increased number of small sized mitochondria and a reduction of the middle and large organelles (fig. 1). Despite the same variations where observed, regarding mitochondrial size population, when hippocampal neurons were pre-treated with CIG, no statistically significant differences were observed between control situation and pre-treated neurons for small and middle sized mitochondria (fig. 1 C and F). About large sized mitochondria, even when the number showed also a reduction, the tendency was different to what observed for H_2_O_2_ treatment; while a sustained reduction of large mitochondrial number was observed for the later, in CIG pre-treated neurons we observed that at 30 min there is an inflection point, indicating that the number of large mitochondria started to increase (fig. 1 J). These results suggest that CIG, through PPARγ activation, is able to prevent the mitochondrial size reduction induced by oxidative stress, protecting neurons from H_2_O_2_-induced mitochondrial altered fusion-fission process.

### Pre-treatment of Hippocampal Neurons with WY, a PPARα agonist, Prevents Mitochondrial Size Reduction Induced by H_2_O_2_ Challenge

**Figure 2:**
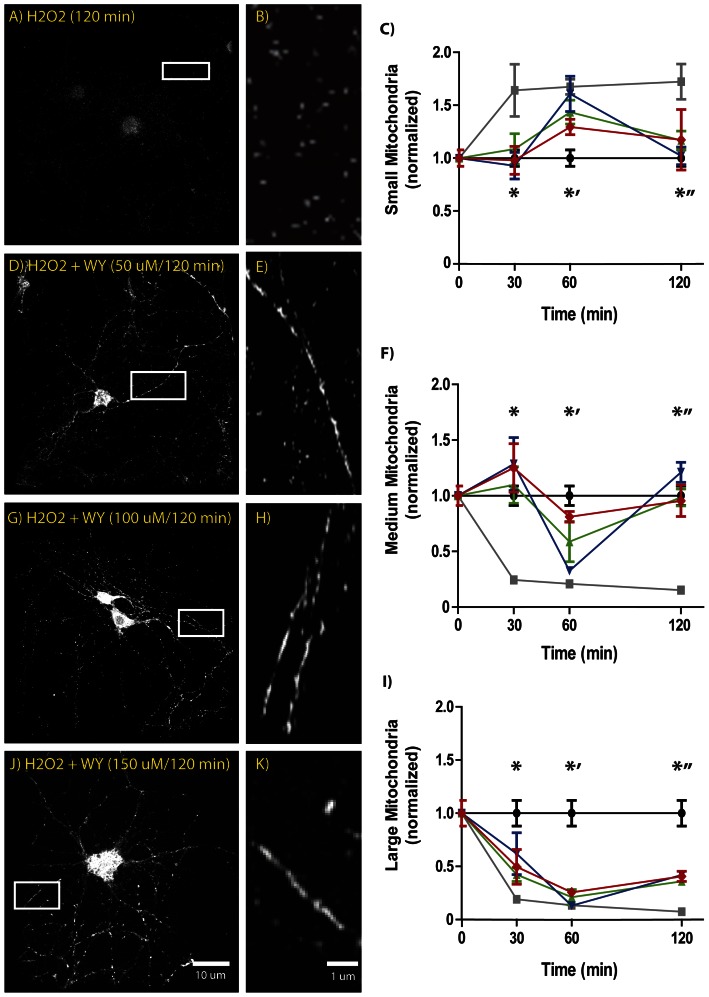
H_2_O_2_-induced mitochondrial size decrease is prevented by WY pre-treatment in hippocampal neurons. A, D, G, J microphotographs of mitochondrial population under different experimental conditions. B, E, H, K: image enlargement to show variations in mitochondrial morphology observed under different experimental conditions. C, F, I: Normalized mitochondrial size quantification (black: control; gray: H_2_O_2_; green: 50 µM WY+H_2_O_2_; blue: 100 µM WY+H_2_O_2_; red: 150 µM WY+H_2_O_2_). C) * indicates statistically significant differences between H_2_O_2_ and Control (*F 9.275, d.f. 4, C.I. 0.06947 to 0.631, t 4.078, p<0.001*); *’ indicates statistically significant differences between H_2_O_2_ and Control (*F 9.275, d.f. 4, C.I. 0.08051 to 0.6564, t 4.183, p<0.001.*), interestingly at 60 min only 100 µM WY shows significant differences respect Control (*F 9.275, d.f. 4, C.I. −0.0009977 to 0.6653, t 3.259, p<0.01)*; *” indicates significant differences between H_2_O_2_ and all other treatments (*F 9.275, d.f. 4. Control C.I. 0.1069 to 0.6828, t 4.482, p<0.001; 50 µM WY C.I. −0.617 to 0.01, t 3.165, p<0.01; 100 µM WY C.I. −0.7046 to −0.06217, t 3.902, p<0.001; 150µM WY C.I. −0.6306 to 0–0303, t 2.969, p<0.05*). F) *, *’ and *” indicate significant differences between H_2_O_2_ and Control (F 21.3, d.f. 4. Control: * C.I. −0.3851 to −0.09656, t 5.457, p<0.001; *’ C.I. −0.3995 to −0.1036, t 5.558, p<0.001; *” C.I. −0.4178 to −0.1218, t 5.961, p<0.001). No significant differences were observed between Control and WY concentrations, except for 100 µM WY at 60 min (*F 21.3, d.f. 4, C.I. −0.386 to −0.04365, t 4.102, p<0.001*). I) At each single time point significant differences were registered between Control and all other experimental conditions; however, at 120 min was possible to observe a different tendency for WY pre-treated curves and that of H_2_O_2_.

Following the same paradigm, when WY was used as pre-treatment on hippocampal neurons for 12 h before H_2_O_2_ challenge, the same preventive effects were observed (fig. 2). Considering the different nature of the WY, we tested three different concentrations in order to properly evaluate the effects of the compound. Once again, H_2_O_2_ treatment alone showed statistically significant differences regarding mitochondrial size compared with the control situation at each time point. On the other hand, WY pre-treatment of hippocampal neurons also prevented mitochondrial size reduction, buffering the toxic effects of H_2_O_2_, suggesting mitochondrial dynamics improvement (fig. 2 C, F and I). Similarly to what was described for the large sized mitochondria under CIG treatment, with WY, the inflection point was reached after 60 min under H_2_O_2_ challenge, and the recovery was statistically significant compared with the H_2_O_2_ treatment alone (fig. 2 I).

### Loss of PGC-1α Signal is Induced under H_2_O_2_ Challenge but Prevented by WY Pre-treatment

**Figure 3:**
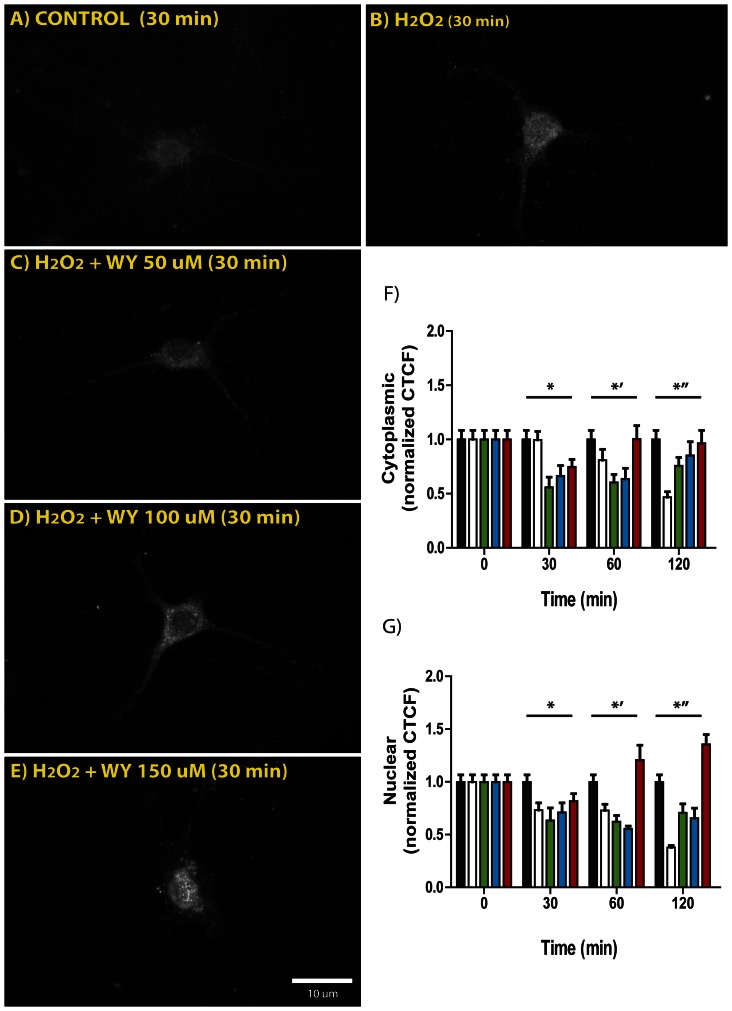
H2O2-induced PGC-1α signal intensity loss is prevented by WY pre-treatment in hippocampal neurons. A–E representative microphotographs of PGC-1α immunodetection in hippocampal neurons. F (cytoplasmic) and G (nuclear), normalized PGC-1α signal intensity quantification by means of the corrected total cell fluorescence (CTCF) (black: control; white: H_2_O_2_; green: 50 µM WY+H_2_O_2_; blue: 100 µM WY+H_2_O_2_; red: 150 µM WY+H_2_O_2_). F) * and *’ indicates statistically significant differences between Control and 50 µM and 100 µM WY (*F 5.131, d.f. 4. 50 µM WY C.I. −0.08866 to −0.007103, t 2.545, p<0.05, and C.I. −0.07239 to −0.000643, t 3.041, p<0.05; 100 µM WY C.I. −0.6401 to −0.001898, t 2.816, p<0.05, and C.I. −0.05095 to 0.02356, t 2.580, p<0.05*). *” indicates statistically significant differences between H_2_O_2_ and Control (*F 5.131, d.f. 4, C.I. −0.07803 to −0.02051, t 5.118, p<0.001)*. G) At 30 and 60 min (* and *’) only 150 µM WY did not show statistically significant differences respect Control situation. Interestingly, at 120 min (*”) also 150 µM WY exhibits significant differences regarding Control, showing an increment of the PGC-1α signal intensity (*F 5.131, d.f. 4, C.I. 0.002515 to 0.09452, t 3.448, p<0.01*).

PGC-1α is localized at both, nuclear and cytoplasmic compartments, preferentially located at the nuclear level [31]; and it expression has been related with enhanced expression of several mitochondrial-related proteins, such as uncoupled proteins (UCP), nuclear respiratory factor (NRF1 and 2) and DRP among others [32], [33]. Fig. 3 shows both nuclear and cytoplasm normalized PGC-1α signal intensity registered by immunofluorescence after H_2_O_2_ challenge. After 30 min a statistically significant signal decreases was observed at the cytoplasm level of hippocampal neurons compared with control situation. Moreover, after 120 min the loss of signal intensity was dramatically lower (*−*50%) at both, cytoplasm and nuclear, levels (fig. 3 F and G). Despite an initial decrease in PGC-1α signal intensity registered after 30 min of H_2_O_2_ challenge, WY pre-treatment was able to prevent PGC-1α signal loss at each single dose; moreover, at 150 µM WY it increased the signal intensity, suggesting PGC-1α increased expression at both cytoplasm and nuclear compartments of hippocampal neurons.

### WY Pre-treatment Enhances Mitochondrial Fission Events in Hippocampal Neurons, Induced by H_2_O_2_ Challenge

**Figure 4:**
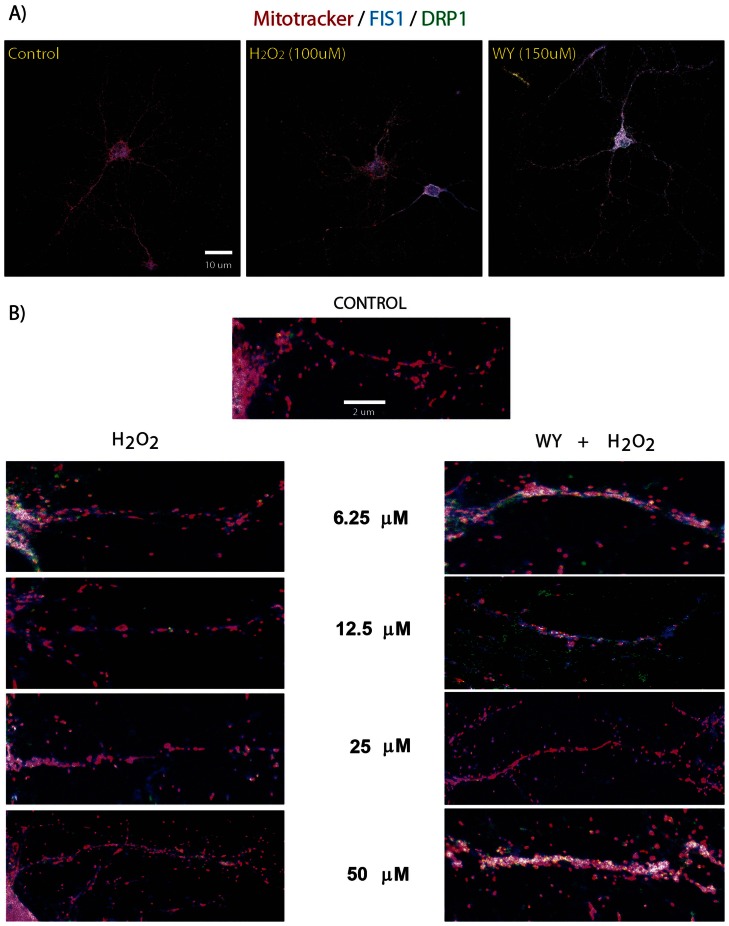
WY pre-treatment induces mitochondrial morphology changes in hippocampal neurons after H_2_O_2_ challenge. A) representative microphotographs of hippocampal neurons showing color merge under specific experimental conditions. B) mitochondrial morphology registered after H_2_O_2_ challenge and with WY pre-treatment.

**Figure 5:**
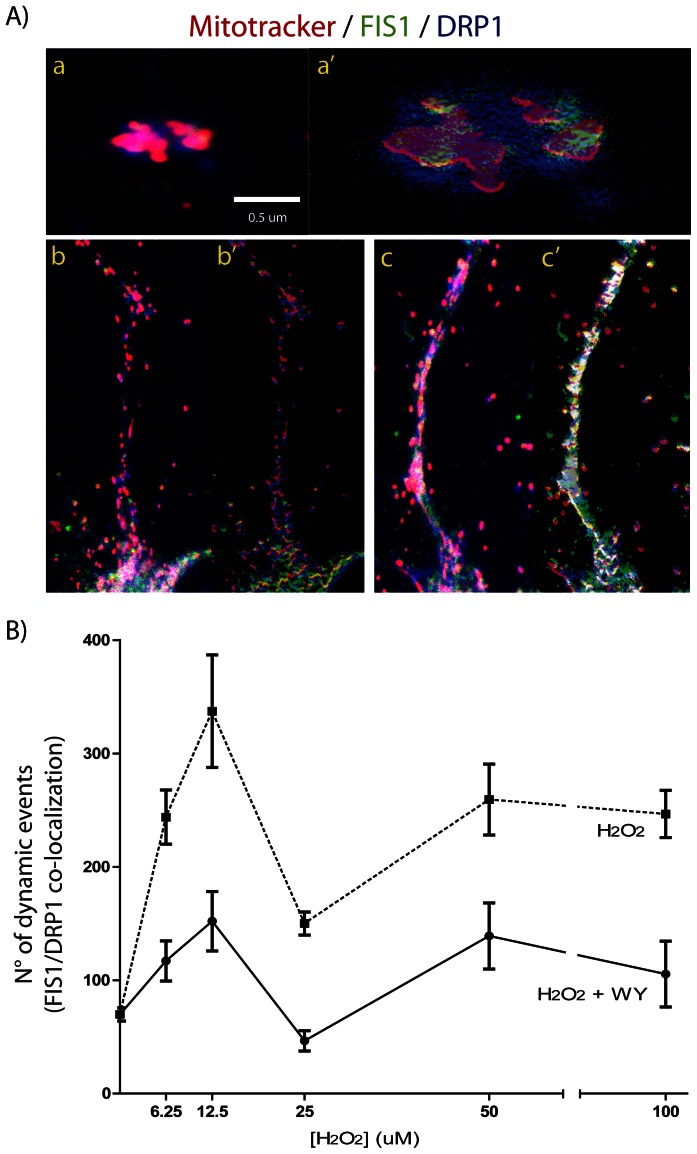
WY pre-treatment increases FIS1/DRP1 co-localization events in hippocampal neurons after H_2_O_2_ challenge. A) microphotograph close up showing localization of FIS1 (green) and DRP1 (blue) on a fissioned mitochondria (a); and on a neuronal projection under H_2_O_2_ challenge alone and with WY pre-treatment (b and c, respectively). Right images were manipulated only to increase color definition (a’, b’ and c’). B) FIS1/DRP1 co-localization quantification. At each H_2_O_2_ concentration statistically significant differences were registered between H_2_O_2_ alone and the WY pre-treated hippocampal neurons (*F 10.36, d.f. 5. 6.25 µM C.I. 40.29 to 213.4, t 4.033, p<0.01; 12.5 µM C.I. 67.02 to 303.6, t 4.311, p<0.001; 25 µM C.I. 0.5261 to 216.5, t 2.765, p<0.05; 50 µM C.I. 2.051 to 238.6, t 2.8, p<0.05; 100 µM C.I. 28.89 to 253.3, t 3.461, p<0.01*). Red channel: mitotracker; Green channel; FIS1; Blue channel: DRP1.

**Figure 6:**
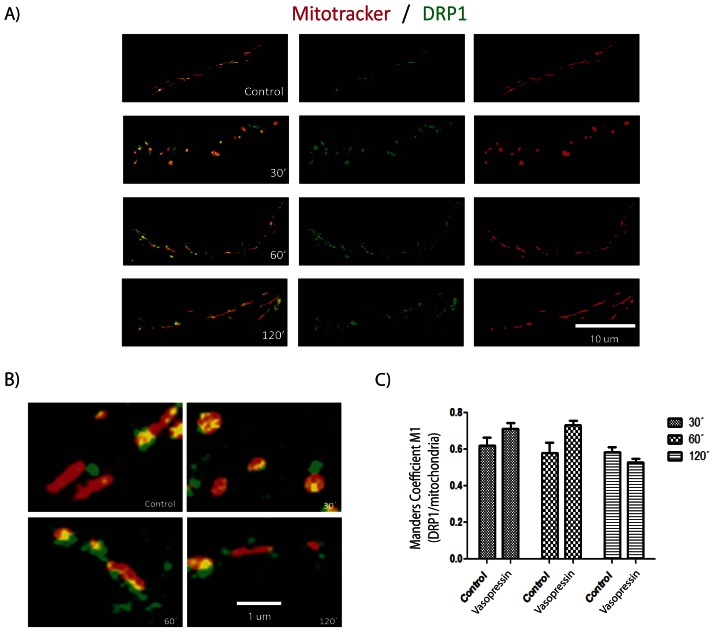
Mitochondrial dynamics events induced after Vasopressin treatment on hippocampal neurons. A) Representative microphotographs of neuronal projections after vasopressin treatment. B) Enlarged images showing Mitotracker and DRP1 signal co-localization registered under different experimental conditions. C) Normalized quantification of signal co-localization.

Mitochondrial size is related with the balance between mitochondrial fusion and fission events [19], [34]. By means of an immunofluorescence assay we establish the mitochondria-related localization of two proteins, DRP1 and FIS1, in order to assess if the H_2_O_2_ challenge and the WY pre-treatment is able to modulate mitochondrial dynamics (fig. 4 and 5). We use several H_2_O_2_ concentrations in order to provide a better overview of mitochondrial response, and the same concentrations were tested after 150 µM WY pre-treatment. As previously observed, the H_2_O_2_ challenge induces a reduction of the mean mitochondrial size in a dose dependent manner, but when hippocampal neurons were pre-treated with WY a recovery of the mean mitochondrial size, close to control situation, was observed (fig. 4 A and B). As expected, H_2_O_2_ treatment alone also induced an increased co-localization of immunofluorescence signals (white spots) suggesting increased fission events (fig. 5 A). Interestingly, the WY pre-treatment induced a higher increase of white spots number in response to H_2_O_2_ challenge, suggesting that WY will favor mitochondrial fission even under oxidative stressors (fig. 5 B). We have also included vasopressin treatment as positive control of a mitochondrial dynamics inducer with well-defined pharmacodynamics [35], [36] (fig. 6). The DRP1 and Mitotracker co-localization induced by vasopressin, allow us to suggest that calcium balance might play a critical role in the modulation of mitochondrial dynamics.

## Discussion

Despite mitochondrial dysfunction has been recognized since long ago as a key event in Aβ-induced neurotoxicity [12], [18], only recently it has been established that mitochondrial dynamics plays a fundamental role in mediating mitochondrial dysfunction [19], [33], [34]. The balance of fusion and fission events is critical in order to maintain healthy mitochondrial population when responding to stressor stimuli [34].

Our results indicate that after H_2_O_2_ challenge, mitochondria undergoes successive fission events without it counterpart, leading to a reduced mitochondrial size. In fact, it has been previously demonstrated that excessive ROS production could damage mitochondrial DNA (mtDNA) leading to mitochondrial malfunction, and that these defective organelles could be rescued through fusion or finally eliminated after fission [34], [37], [38]. These findings suggest, and allow us to hypothesize, that the cell antioxidant activity as well as the mitochondrial metabolic capacity were exceeded by the H_2_O_2_ challenge, and resulted in mitochondrial damage without any recovery response. Moreover, the final loss of PGC-1α signal registered after H_2_O_2_ challenge serves to offer further support to this hypothesis. Even when described initially just as a cold inducible factor [39], further investigations have positioned PGC-1α, as a critical modulator of several metabolic processes and as a key element of mitochondrial biogenesis [32], [40]. In fact, PGC-1α activity linked to PPARs, RXR, and estrogen related receptor α (ERRα) have been related with the expression of several mitochondrial proteins, such as UCP, NRF1, NRF2, DRP1, FIS1, MFN2 [19], [26], [32], [40], [41]. In consequence, the loss of PGC-1α signal observed in our work suggest a diminished expression rate of several mitochondrial proteins related with fusion-fission events affecting negatively mitochondrial dynamics and leading to mitochondrial dysfunction.

Interestingly, it was possible to observe that PGC-1α loss does not occurs immediately but after 30 min H_2_O_2_ challenge. Despite this finding could results confuse, it has been described that H_2_O_2_ is able to induce an initial cell response linked to increased antioxidant enzymes expression and enhanced mitochondrial activity [42]. In fact, it was possible to observe that even as very low concentration, such as 6.25 µM, H_2_O_2_ is able to induce increased mitochondrial fission events that could reflects this initial cell response. Moreover, it seems to be a critical concentration (25 µM) at which the H_2_O_2_ challenge induces less fission events than lower and higher concentrations, suggesting that this point could indicate the change between a physiological compensatory mitochondrial response and a pathological outcome after a stressor stimuli. Furthermore, this phenomenon might by relate with the physiological role often described for ROS on several cell processes [42]–[44].

On the other hand, we used two different PPAR agonists in order to assess the protection derived of the PPARs pre-treatment. The therapeutic potentialities of PPARs on several disorders have been widely assessed by our research group as well as others [20]–[24], [45]–[50]. Despite we have previously described PPARγ-related mitochondrial protection [22], here we describe for the very first time the relation of PPARγ agonist with mitochondrial dynamics. In fact, the CIG pre-treatment protects mitochondria from size reduction, suggesting the modulation of fusion-fission events. In the same way, WY, a PPARα agonist, also exhibits the same property. If we consider that main effects were observed after 30 min H_2_O_2_ challenge, it is possible to hypothesize that initially there was an increased expression of antioxidant enzymes, enhancing cell antioxidant capacity, delaying the mitochondrial response to the stressor stimulus. Furthermore, we have recently demonstrated that 4-phenylbutyrate and WY are able to prevent and rescue cognitive impairment in a double transgenic mouse model of AD [24]. Even when these two drugs acts as different agonists (PPARγ and PPARα, respectively), the results obtained for both treatments were quite similar. According to available information a common point for the mitochondrial dynamics modulation of these drugs will be the activation of the PGC-1α [51], [52].

As expected, WY pre-treatment not only prevents the reduction of mitochondrial size, but also increases the PGC-1α signal intensity at the cytoplasm and nuclear level, suggesting that WY is able to induce the expression of PGC-1α. Moreover, the higher number of fission events obtained under WY pre-treatment, together with the increased mitochondrial size, suggests enhanced mitochondrial dynamics events, derived from PGC-1α increased expression, inducing fusion and fission as a coordinated process, and that could be at the basis of the neuroprotective mechanism observed for these drugs. Interestingly, the number of fission events was higher than registered for H_2_O_2_ alone, suggesting that mitochondrial fission should not be considered as a negative event but as a critical step of mitochondrial physiology which allows better response to stressor stimuli. Our results, together with previous findings that have linked the increased ROS production due to Aβ peptide [21], [53], allow us to consider that this mechanism offers new and interesting perspectives in ROS-related neurodegenerative disorders, such as AD. On the other hand, we have recently conducted several experiments using vasopressin, a known hormone that has shown to induce mitochondrial dynamic modulation. Intracellular calcium balance lies at the basis of vasopressin, mainly through a direct action on internal calcium reserves, such as endoplasmic reticulum, and/or inducing extracellular calcium influx through voltage-dependent calcium channels (VDCC) [35]. Interestingly, both PPARs, α and γ, have been linked with calcium balance [54], [55]. Moreover it is well known that increased Ca^2+^ levels, mainly through VDCC, leads to activation of protein kinase C (PKC) and further phosphorylation of DRP1 [36]. Considering our results, we could hypothesize that also part of the effects observed on mitochondrial dynamics might be related with the PPAR-mediated intracellular calcium balance, affecting mitochondrial dynamics-related proteins activity.

**Figure 7:**
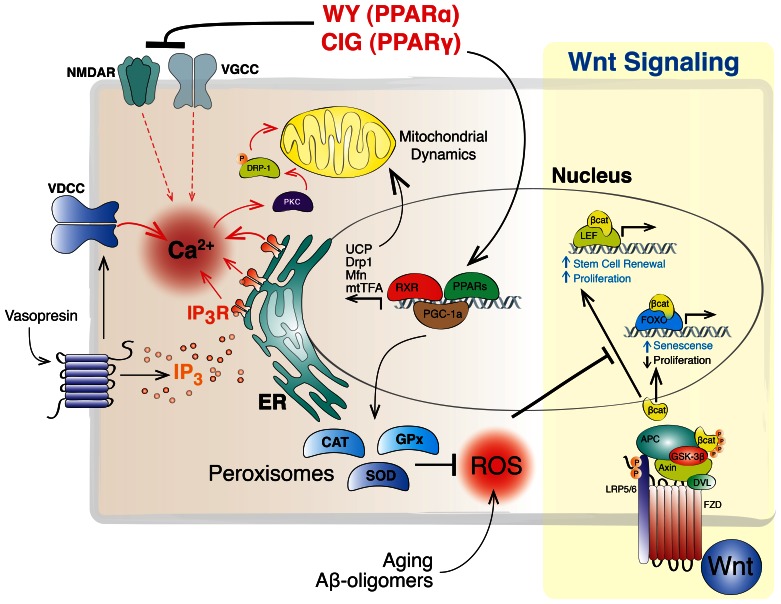
PPAR-mediated mitochondrial dynamics modulation. We have previously proposed a direct link between antioxidant PPARs activity and Wnt signaling pathway [19]. Based on our present results we hypothesize that PPARs are able to modulate mitochondrial dynamics trough different cellular mechanisms. Complementary to the Wnt signaling pathway (*FZD*, frizzled; *LRP 5/6*, low density lipoprotein receptor-related protein; *DVL*, disheveled; *βcat*, β-catenin), PPARs might act inducing directly the expression of several mitochondrial-related proteins (*UCP*, uncoupled protein; *DRP1*, dynamin related protein 1; *Mfn*, mitofusin; *mtTFA*, mitochondrial transcription factor). Additionally, the PPARs-mediated calcium balance might also offer another control point for mitochondrial dynamics modulation (*VDCC*, voltage dependent calcium channels; *NMDAR*, NMDA receptor; *VGCC*, voltage-gated calcium channels; *PKC*, protein kinase C).

Despite valid concerns surrounds PPARs, overall related to it toxicity and carcinogenic effects derived from it use [56], the therapeutic opportunities offered by this family of molecules is out of discussion [4], [57]. However, the mechanism involved in the wide range of beneficial effects observed for PPARs agonists are quite unknown, and even when several studies have offered some possible explanations for part of these effects, they have failed to explain others. Years ago, we have proposed a direct link between PPARs and the Wnt signaling pathway [58] at the basis of several neuronal mechanisms. Here we propose an additional mechanism relating PPARs agonist and mitochondrial dynamics (fig. 7), and how this relation could act as a mitochondrial protective mechanism that finally will confers enhanced metabolic protection to hippocampal neurons against oxidative stress.
